# Neonatal Hearing Screening: failures, hearing loss and risk indicators

**DOI:** 10.1590/S1808-86942011000600015

**Published:** 2015-10-19

**Authors:** Raquel Mari Onoda, Marisa Frasson de Azevedo, Amélia Miyashiro Nunes dos Santos

**Affiliations:** 1 MSc, Speech and Hearing Therapist; 2 PhD; Professor at the Department of Speech and Hearing Therapy; 3 PhD; Professor at the Department of Pediatrics

**Keywords:** hearing, hearing loss, infant, newborn, hearing tests, neonatal screening

## Abstract

**Abstract:**

To check the rate of failure, hearing loss and its association with demographic variables and risk indicators for hearing loss in newborns submitted to the Newborn Hearing Screening in a secondary hospital.

**Materials and Methods:**

Cross-sectional and retrospective study, involving 1,570 newborns submitted to the different stages of the Newborn Hearing Screening Program. Initially, we carried out otoacoustic emission tests (ILO Echocheck) and the cochlear-eyelid reflex. Afterwards, we analyzed the demographic and clinical characteristics of the newborns, screening rate of failure, hearing loss and its association with demographic variables and risk indicators.

**Results:**

Twenty-six newborns had failures in the first stages of the Program (1.7%), who were then referred to diagnostic evaluation. Of these, 16 (61.5%) did not come, two (7.7%) had normal results and eight (30.8%) were diagnosed with hearing disorders. The screening failure rate was 1.7% and the frequency of hearing disorders was 0.5%.

**Conclusions:**

Pre-term newborns of very low birth weights had higher rates of screening failures and a greater occurrence of hearing changes. The factors associated with screening failure and hearing changes were similar to the ones described in the literature.

## INTRODUCTION

Hearing loss is one of the most common sensorial disorders. A clinically significant hearing loss may affect 1-3 for every 1000 low-risk newborns^1^,^2^; and among newborns in the ICU this rate can reach 2% to 4%^3^,^4^.

After analyzing the literature, COMUSA (Multidisciplinary Committee on Hearing Health), made up by otolaryngologists, pediatricians, speech and hearing therapists, and other professionals, made 21 recommendations for early identification, diagnosis and treatment of newborns and infants with hearing impairment^5^.

In the present study we tried to analyze the demographic and clinical characteristics of the newborns who participated in the Neonatal Hearing Screening Program in a secondary level maternity, identifying the level of failure and the occurrence of hearing loss and its association with the following variables: gestational age, birth weight and neonatal clinical occurrences.

## MATERIALS AND METHODS

This was a retrospective study in which we collected the results from the Neonatal Hearing Screening Program implemented in the Neonatal Unit of a Municipal Hospital located in the north of the city of São Paulo, SP. The annual number of live births, seen in the Neonatal Unit in this hospital is around 1,500; with rates of low-birth weight (birth weight <2,500g) of 11%; very low birth weight (birth weight <1,500g) rate of 1.6% and of extreme low birth weight (birth weight <1000g) rate of 0.8%.

In the study, we included the newborns whose hearing screenings were done by the author, during 20 months, between February of 2004 and December of 2006.

This project was submitted to and approved by the Ethics in Research Committee under protocol # 1332/06.

The Neonatal Hearing Screening (NHS) was carried out by means of Transient Stimulus Evoked Otoacoustic Emissions (TEOE) and the Cochleo-eyelid Reflex (CER) by means of an agogô musical instrument (large campanula) at 100 dBSPL of intensity.

TEOE was carried out in both ears using the ILO EchocheckT system, a portable device which uses click stimuli involving frequency bands between 1,500 Hz and 3,800 Hz. The click is presented at an intensity of 75 to 83 dBpeSPS. The response was considered positive (pass) when the otoacoustic emissions captured were 6 dB higher than the noise.

CER happens in 100% of normal hearing children and its lack suggests bilateral hearing loss or central disorder. The study is carried out with an intense sound stimulus and the response is considered present when there is a contraction of the eye's orbicular muscle, seen by eyelid movement.

The newborn who failed the screening was submitted to a complete audiological evaluation for diagnostic purposes. The assessment was based on the study of the TEOE, acoustic immittance measures obtained by the AZ7 immittance meter with a 226 hertz probe for the tympanometric curve, behavioral assessment and brainstem Hearing evoked potential (BAEP), with the Navigator Pro-Biologic Systems Corp^®^ device with a click-type stimulus.

The children who had risk indicators for hearing impairment, even those who passed the hearing screening, were referred to Hearing development follow up.

The hearing loss was classified as conductive when associated with middle or external ear disorders; or sensorineural, associated with cochlear disorders classified as mild to profound degree^6^, or retrocochlear, because of a hearing neuropathy or neural conduction changes in the brainstem Hearing pathways.

As far as the demographic characteristics of the children included in the study are concerned, we observed the following data: newborn gender, birth weight, gestational age and Apgar score.

Concerning hearing impairment risk analysis, we collected the following variables: consanguinity, congenital malformations, perinatal asphyxia, hyperbilirubinemia, peri-intraventricular hemorrhage, meningitis, seizures, need for mechanical ventilation, use of ototoxic medication, congenital infections such as syphilis, rubella, toxoplasmosis, cytomegalovirus and human immunodeficiency virus (HIV) diagnosed during gestation or in the neonatal period, smoking, alcohol drinking or the use of illegal drugs during pregnancy; family with hearing loss, diagnosis or suspicion of genetic syndromes made by the pediatrician and/or geneticist, duration of neonatal ICU stay.

We analyzed demographic and clinical characteristics of the newborns included in this study and the rate of failure in the screening, the occurrence of hearing loss and an association between failure in the test and gestational age, birth weight and the main neonatal complications.

The results obtained were described as mean and standard deviation of the numerical variables and frequency for the categorical ones. The investigation concerning the factors associated with otoacoustic emission test failure was carried out by means of a univariate analysis, using the chi-squared or Fisher's test, considering *p*<0.05 as significant.

## RESULTS

During the study, 4,593 children were born at the maternity of this hospital. The NHS was carried out in 1,805 newborns (39.3%), 1,615 (89.5%) were born at term, 146 (8.1%) were preterm with birth weight higher than 1,500g and 44 (2.4%) preterm with birth weight lower than 1,500g.

**Figure 1:**
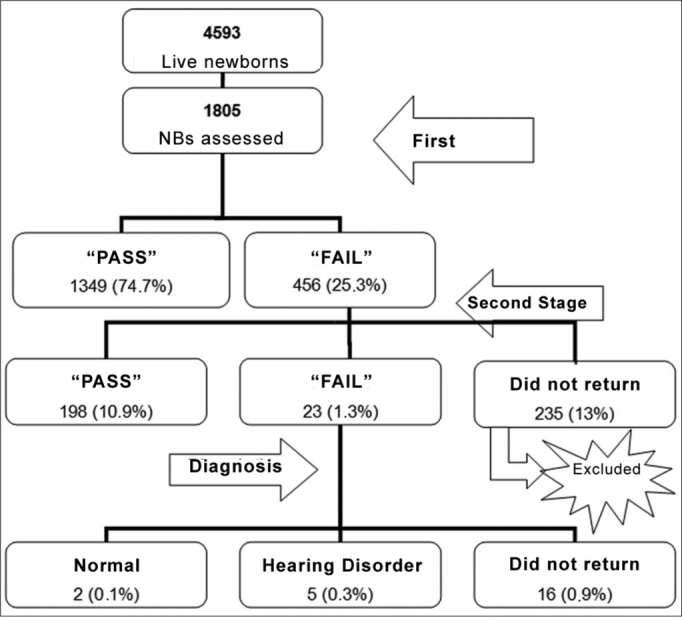
Number and percentage of children considered in the Newborn Hearing Screening Program of the Vereador José Storópolli Hospital.

Figure 1 represents the number and percentage of children who participated in the different stages of the Newborn Hearing Screening Program carried out in the present study.

Of the 1,805 newborn evaluated, 235 did not come for the second stage of the NHS program; thus, only 1,570 newborns were evaluated.

The 235 newborns who did not return, were compared to the 1,570 newborns of the study in relation to gestational age, birth weight and risk indicators, with the goal of checking for similarities between the groups for statistical purposes.

The newborns who completed the second stage of the NHS had similar characteristics in relation to gestational age (38.8±2.2 versus 38.7±2.1 weeks, *p*=0.821) and birth weight (3,107±600 versus 3,169±587 grams, *p*=0.140), when compared to the ones who did not return.

Thus, we concluded that the children who did not return for an assessment at the second stage were similar to the ones who continued in the NHS program, except for the greater frequency of peri-intraventricular hemorrhage (1.7% versus 0.4%, *p*=0.031*).

The sample studied was made up of 1,570 newborns, 790 (50.3%) females and 780 (49.7%) males; 1,401 (89.2%) born at term and 169 (10.8%) were premature.

The children without TEOE and/or CER were considered as failure in this study. Therefore, the failure rate in this study's sample was 26 (1.7%). Of these, nine (0.6%) had one or more risk indicators for hearing loss.

The mean gestational age and birth weight of the children in relation to the NHS are depicted on Table 1.

**Table 1:** Demographic characteristics of the 1,570 newborns studied, according to their NHS results.

		n	Mean	Standard Deviation	Maximum	Minimum	Mode	Median	*p*-value
Gestational age (weeks)	FAILED	26	36.2	4.6	41.1	24	38.4	37.9	0.133
	PASSED	1521	39.1	9.5	42.9	32	40	39.1	
Birth weight (grams)	FAILED	26	2516	1034	4290	850	2350	2847.5	0.007*
	PASSED	1536	3117	586	5070	2018	3000	3200	

n=number

In regards of risk indicators for hearing impairment, 1,349 (85.9%) newborns, of the 1,570 included in the present study, did not have any risk indicator; and 221 (14.1%) had one or more risk indicators.

In order to assess the failure rate at NHS in relation to the variables: gender, prematurity, ear tested, stay in newborn ICU, risk factors for hearing impairment, we considered the 1,570 newborns who made up the series investigated in this study.

The failure rate was not related to the child's gender (*p*=0.223) and ear side (*p*=1.000). There was a greater failure rate among preterm newborns (4.1%) in relation to those who were born at term (1.4%); *p*=0.017.

In order to analyze failures in relation to the gestational age and birth weight, the children were broken down into three groups:

• At term Group (TG): at term newborns with birth weight equal to or higher than 1,500g.
• Preterm Group (PT ≥1,500g): premature babies with birth weight equal to or higher than 1,500g.
• Preterm with very low birth weight Group (PT MBP): premature babies with birth weight lower than 1,500g.

The rate of NHS failures in the PT MBP (15.0%) group was higher than that from the GT (1.2%) and PT >1,500g (1.6%) groups, *p*<0.001.

In order to analyze the influence of risk indicators concerning hearing impairment, the sample was broken down according to the number of risk factors presented (from one to five).

Table 2 depicts the failure rate in the neonatal hearing screening in relation to the number of risk indicators. After employing Fisher's exact test, we noticed a statistically significant association between NHS failure and the number of risks. Failures happened less often in children with a maximum of one risk indicator, when compared to those who had two or more risk indicators.

**Table 2:** Results from the NHS, according to the number of risk indicators for hearing impairment.

Risk #	Passed	Failed	Total
Zero	1332 (98.7%)	17 (1.3%)	1349 (100.0%)
1	98 (98.0%)	2 (2.0%)	100 (100.0%)
2	65 (95.6%)	3 (4.4%) *	68 (100.0%)
3	40 (95.2%)	2 (4.8%) *	42 (100.0%)
4	8 (80.0%)	2 (20.0%) *	10 (100.0%)
5	1 (100.0%)	(0.0%)	1 (100.0%)
Total	1544 (98.3%)	26 (1.7%)	1570 (100.0%)

*p*-value: 0.003

We analyzed 15 risk indicators for HL. Table 3 depicts the statistical analysis and p-values for each risk indicator associated with the failure in neonatal hearing screening, which are depicted on Table 3.

**Table 3:** NAS results according to the risk indicators analyzed.

	Passed	Failed	*p*-value
Family history	38 (2.5%)	2 (7.7%)	0.146
Consanguinity	11 (0.7%)	0	>0.999
Congenital infections	42 (2.7%)	0	0.517
Craniofacial malformation	8 (0.5%)	3 (11.5%)	0.001*
Hyperbilirubinemia	1 (0.1%)	1 (3.8%)	0.033*
Mechanical ventilation	39 (2.5%)	3 (11.5%)	0.030*
Drugs or alcohol during pregnancy	7 (0.4%)	0	>0.999
Ototoxic	46 (3.0%)	3 (11.5%)	0.035*
Asphyxia	13 (0.8%)	1 (3.8%)	0.209
Seizure	3 (0.2%)	0	>0.999
Meningitis	4 (0.3%)	0	>0.999
Syndromes	4 (0.3%)	0	>0.999
Peri-intraventricular hemorrhage	4 (0.3%)	2 (7.7%)	0.003*
ICU > 48 hours	134 (8.7%)	6 (23.1%)	0.003*
Very low birth weight	32 (2.1%)	6 (23.1%)	<0.001*

### Hearing loss index according to demographic variables and risk indicators

After diagnostic assessment, two children (0.1%) had normal results, which were considered false-positives. 16 (1%) children did not show up for their return visit and eight (0.5%) had a hearing impairment diagnosis - four being sensorineural hearing loss (0.25%), one conductive hearing loss caused by craniofacial malformation (0.06%); and three children with suspected retrocochlear disorder (0.2%).

The hearing impairment frequency analysis was similar for both genders (*p*=0.625) and it was significantly higher in pre-term babies (3.6% vs. 0.2%, *p*<0.001), when compared to their term newborn counterparts. Among premature babies, such disorder was more frequency among those of very low birth weight, when compared to those which birth weight higher than 1,500g (12.8% vs. 0.8%, *p*<0.001).

We noticed a higher occurrence of hearing disorders in newborns who had one or more risk indicators (Table 4). Nonetheless, it is worth stressing that one child diagnosed with hearing loss did not have any risk indicator.

**Table 4:** Hearing disorders in relation to the risk indicator present.

Risk #	Normal	Hearing disorders	Total
Zero	1335 (99.9%)	1 (0.1%)	1336 (100.0%)
1	98 (98.0%)	2 (2.0%)	100 (100.0%)
2	64 (98.5%)	1 (1.5%)	65 (100.0%)
3	40 (95.2%)	2 (4.8%)	42 (100.0%)
4	8 (80.0%)	2 (20.0%)	10 (100.0%)
5	1 (100.0%)	0	1 (100.0%)
Total	1546 (99.5%)	8 (0.5%)	1554 (100.0%)

*p*-value: 0.001*

Table 5 depicts the risk indicators associated with hearing disorders (Table 5). In this analysis we took off the 16 newborns who did not complete the diagnosis process; thus, we analyzed 1,554 children, from whom 1,546 did not have any hearing disorder and eight had a hearing disorder.

**Table 5:** Analysis of the children with hearing disorder, according to the presence of risk indicators.

	Normal	Hearing disorder	*p*-value
Family history	39 (2.5%)	1 (12.5%)	0.017*
Consanguinity	11 (0.7%)	0	0.945
Congenital infections	43 (2.8%)	0	0.798
Craniofacial malformation	8 (0.5%)	1 (12.5%)	0.046*
Hyperbilirubinemia	1 (0.1%)	0	0.995
Mechanical ventilation	37 (2.4%)	4 (50.0%)	<0.001*
Drugs or alcohol during pregnancy	7 (0.4%)	0	0.964
Ototoxic drugs	45 (2.9%)	4 (50.0%)	<0.001*
Asphyxia	14 (0.9%)	0	0.930
Seizures	3 (0.2%)	0	0.985
Meningitis	4 (0.3%)	0	0.980
Syndromes	4 (0.3%)	0	0.980
Peri-intraventricular hemorrhage	3 (0.2%)	2 (25.0%)	<0.001*
ICU stay > 48 hours	137 (8.9%)	5 (62.5%)	0.003*
Very low birth weight	24 (1.6)	6 (75%)	<0.001*

There was a statistically significant association between hearing disorders and the risk indicators: family history of hearing loss, craniofacial malformation, use of mechanical ventilation, use of ototoxic drugs, peri-intraventricular hemorrhage, ICU stay for more than 48 hours and very low birth weight.

## DISCUSSION

Newborn Hearing Screening provides for the early detection of hearing disorders, thus enabling intervention before six months of age^7^.

A hearing health program must bear four stages: detection or hearing screening, audiological diagnosis, hearing aid fitting and the intervention of an audiologist - expert in educational audiology^7^.

Newborn Hearing Screening is considered a process, and not an event, which provides parents and children a follow up, from pre-screening instructions all the way to the treatment and follow up of the child diagnosed with the hearing loss and the child's family^8^. Nevertheless, there are still many challenges to be faced, such as: Implementation of a Newborn Hearing Screening Program with the help of maternities - both administratively and also considering the professionals involved in newborn care; effective follow up of those babies who failed the screening process or those bearing risk indicators for hearing disorder. Thus, we can reduce the large number of babies who fail to report back and thus missing the opportunity for a late diagnosis.

Another big challenge is the proper intervention and selection of hearing aids, besides speech and hearing therapies to promote proper language development^8^.

Only 39.3% of the 4,593 babies born during the study period participated in the Newborn Hearing Screening Program. This rate of coverage was lower than 95% of what was recommended to consider for an effective Newborn Hearing Screening Program^3^,^4^.

National and international studies report NHS coverage varying between 41.6% and 99.2%^9^, ^10^, ^11^, ^12^, ^13^.

In order to guarantee a hearing screening for all newborns, it would have to be performed every day of the week by a trained team concerning the equipment used as well as the protocol to be followed. Moreover, the NHS program should include pediatricians, otolaryngologists and expert audiologists^14^. Facts which did not happen in the present study, because the NHS was done in only two periods per week and by one professional only.

Of the screenings carried out in the present study, 74.7% had a “passing” result in the first stage of the screening program.

The rate of failure in the NHS (25.3%) for newborns with and without risk indicators for hearing impairment was higher than those in some studies present in the literature^10^,^13^,^15^. In 2007, the *Joint Committee on Infant Hearing* (JCIH), recommended that failures in the first stage should not surpass 10%. Such result may be explained by the age in which the first NHS was carried out, since most of the children were assessed within 48 hours of life, before being discharged from the hospital.

Despite the possibility of capturing TOAEs already after 24 hours of life, it is also known that middle ear effusion is also very common in the first 48 hours of life^16^.

Moreover, factors such as an excess of environment noise or routine procedures which make the child more restless (bath, handling for an exam, drug administration), the external auditory meatus being obstructed by vernix or by wax may contribute to the high rates of retests in NHS programs^17^.

The high rate of evasion found in the present study and also described in the literature is still a major challenge for audiology professionals^9^,^12^,^18^. The socioeconomical conditions of the population seen could be one of the factors associated with the high rate of newborn evasion in the second stage of the NHS. The high rate of evasion in the diagnostic stage could also be explained by the difficulty in access, having seen that the diagnosis was made in another hospital, far from the place of birth.

There is a huge lack of information for parents and the professionals who care for these newborns concerning the importance of NHS in the early detection of congenital hearing disorders and of late or progressive onset^3^,^18^.

There has been a great difficulty concerning the mothers' understanding of the importance of NHS in the early diagnosis and treatment of babies with hearing disorders. Many mothers have reported financial difficulties in commuting to the hospital for the follow up visits.

We must stress that our hospital cares for many teenage mothers, some with difficulties in coping with the situation of an unplanned pregnancy and without much interest in NHS. We also have a lot of immigrant patients whom, besides having the difficulties aforementioned, also have some worsening factors such as: communication challenges, some illegally living in the country and who do not return to the hospital in fear of being identified.

Thus, studies which have focused the causes for high rates of evasion, considering the social and demographic factors of the population considered, are needed in order to overcome this challenge.

According to the Brazilian Committee on Hearing Loss During Childhood (1999) and the *JCIH* (2007), the rate of false-positives should not be higher than 3.0% in relation to the total number of children assessed. The rate of false-positives found in the present study (0.1%) is within recommended levels.

From our sample, 221 newborns (14.1%) had one or more risk indicators, and such index was similar to the ones found by other authors^19^.

The most frequent risks found were similar to the ones found in the literature. Risk indicators associated with ICU stay^11^, use of ototoxic drugs^11^,^12^,^19^,^20^, congenital infection^11^, mechanical ventilation^11^,^12^,^19^ and family history of hearing loss^11^,^20^ were the most frequent risk indicators found in the literature.

The percentage number of children who failed and were referred to diagnosis was 1.7%. Such figure is in agreement with what is recommended by the Brazilian Committee on Hearing Loss (1999) and the *JCHI* (2007), which suggest that such index should not be higher than 4% of the individuals assessed.

Based on the publications compiled, we noticed that the percentage of failures can be impacted by many factors: environment noise or baby noise during the screening itself, sleepiness or awareness state of the newborn, examiner's experience with the equipment being used, time of NHS program existence, number of newborns assessed, protocol and procedure utilized, besides the demographic traits of the newborn, complications and risk indicators for hearing impairment, which will be discussed below^9^, ^10^, ^11^,^13^.

In the present study, there were no statistically significant differences in the gestational age mean value among the children who passed and those who failed the NHS.

Now, birth weight was a relevant variable. The children who failed the NHS had the lowest mean weight.

Many studies have shown associations between birth weight and NHS failure and/or hearing disorders^12^,^21^. Frequently, very low birth weight newborns, have many risk indicators. These are children born asphyxiated, who needed mechanical ventilation for a long period of time, they are given ototoxic antibiotic treatment and are subject to infection and/or meningitis^22^.

As far as the gender variable is concerned, we did not find significant differences, which is in agreement with some studies in the literature^23^.

Concerning the ears tested, failures in the newborn hearing screening were similar in both ears, which are different from what was obtained from one of the compiled studies^24^, which found a greater occurrence of failures in the left ear.

Comparing the frequency of failures in the newborn hearing screening program among babies born at term and pre-term, a higher rate of failures was found among pre-term newborns, a result which was similar to the ones found in the literature studied^21^,^25^.

In the present study, besides the gestational age, birth weight was also important considering NHS failures. The statistical analysis showed that the rate of failures was higher among those pre-term babies with very low birth weight. Many studies have reported this same finding^21^.

Failures in the newborn hearing screening program were the most prevalent in the group of children with the most risk indicators.

Numerous studies have described that the more risk indicators a person has, the higher will be the likelihood of this person developing some sort of hearing disorder^25^,^26^.

Risk indicators: cranial malformation, hyperbilirubinemia, mechanical ventilation, use of ototoxic drugs, peri-intraventricular hemorrhage, ICU stay longer than 48 hours were variables associated with NHS failure.

Craniofacial malformation has been considered a risk factor associated with hearing disorders^25^.

It is known that hyperbilirubinemia is toxic to the hearing apparatus and to the central nervous system, and it can cause sequelae such as hearing loss, hearing neuropathy and encephalopathy. Numerous studies have described the correlation between hyperbilirubinemia and hearing disorders^20^,^27^.

Risk indicator: mechanical ventilation, also seems to be associated with hearing disorders in the literature^28^.

The effect of ototoxic drugs in the hearing of high-risk newborns has been compared by many authors^20^,^28^,^29^.

Studies have showed the presence of peri-intraventricular hemorrhage in children with hearing disorders^20^ and some authors have described it as one of the indicators associated with hearing neuropathy^30^.

The greater need to send to the ICU those newborns who failed NHS shows that such babies have a greater risk of failing NHS, since, in general, they have other factors associated^25^,^31^.

The rate of hearing disorders, considering the 1,554 babies who completed the NHS stages and the audiologic diagnostic was 0.5%. The hearing loss rate in the present study was similar to that reported by studies carried out in populations with similar traits to the ones studied^10^, ^11^, ^12^, ^13^,^15^,^19^,^20^.

There were no differences between males and females insofar as the diagnosed hearing disorders are concerned. However, in relation to gestational age and weight at birth, it has been noticed a greater percentage of very low birth weight premature babies with hearing disorders. Some studies have reported a greater rate of hearing loss in premature children^21^. Many studies have reported that birth weight below 1,500g is one of the most frequent indicators associating hearing disorders with children^6^,^12^,^20^,^30^.

It is worth stressing the importance, not only of doing the NHS in premature babies, but also to follow up on their hearing and language development. Premature children may present a delay in their Hearing behavior when compared to their counterparts born at term^32^. Children with a positive family history for hearing impairment during childhood must be considered at risk for progressive and/or late hearing loss^4^.

In regards of the number of risk indicators presented by the group of term newborns diagnosed with hearing loss, one child did not have any risk factor, and another one had craniofacial malformation; in the group of premature babies with birth weights higher than 1,500g, the child diagnosed with sensorineural hearing loss had only the risk of family history for HL; and in the group of premature babies with birth weight below 1,500g, besides the very low birth weight risk, all the children diagnosed with hearing disorders remained in the ICU for a period longer than 48 hours, four needed mechanical ventilation, four used ototoxic drugs and two had peri-intraventricular hemorrhage. A proper interview made by the NHS team is fundamental to identify the risks associated with hearing loss and the need to follow these children up, even if they had normal results in the NHS.

It is recommended that all children who spent over five days in the ICU be referred to hearing assessment by means of the BAEP, in order to detect children with hearing neuropathy spectrum.

Many studies have shown an association between hearing disorders and risk indicators for hearing loss^6^,^33^.

Although children with a risk for hearing loss have a greater likelihood of having disorders, there are a considerable number of children diagnosed with hearing loss who do not have any risk factors. In the present study, one of the eight children diagnosed with hearing disorders did not have any risk factor.

Compiled studies in the literature report significant rates of children diagnosed with hearing loss who did not have risk indicators for hearing disorders^33^.

There was a statistically significant association among risk indicators for hearing disorder: family history for hearing loss, craniofacial malformation, need for mechanical ventilation, use of ototoxic drugs, peri-intraventricular hemorrhage, ICU stay for over 48 hours and very low birth weight.

Based on the results found in the present study we can make many suggestions to the NHS team. Besides being a well-trained and well-structured team, it must me committed to performing NHS every day of the week, have a proper and effective follow up of these babies who failed NHS and those who had risk indicators for hearing loss and must invest in practice and experience in order to increase the efficacy of the Program^10^,^33^.

The importance and implementation of the NHS programs in Brazil has grown substantially; however, there are numerous challenges preventing it from being effective, especially in maternities which serve the low income population, since the rate of evasion among this population during the NHS program is high. Studies which analyze the best method to bring mothers awareness about the importance of the audiological diagnosis and the minimization of evasion factors in the NHS process are valid in order to make the NHS program more effective.

## CONCLUSIONS

1 The rate of detected failures by otoacoustic emissions and the use of the cochleo-eyelid reflex was 1.7%;
2 The prevalence of hearing disorders found in the present study was 0.5%;
3 The very low birth weight newborns have higher newborn hearing screening failure rates and hearing disorders;
4 The failure rate in newborn hearing screening was associated in a statistically significant fashion with the risk indicators: craniofacial malformation, hyperbilirubinemia, need for mechanical ventilation, use of ototoxic drugs, presence of peri-intraventricular hemorrhage, stay in middle to high risk the neonatal ICU for more and 48 hours and birth weight below 1,500g;
5 Hearing disorders were significantly associated with the variables: family history for hearing loss, craniofacial malformation, use of ototoxic drugs, need for mechanical ventilation, presence of peri-intraventricular hemorrhage, stay in middle to high risk neonatal ICU for over 48 hours and birth weight below 1,500g.
